# Mapping interactions between the CRAC activation domain and CC1 regulating the activity of the ER Ca^2+^ sensor STIM1

**DOI:** 10.1016/j.jbc.2022.102157

**Published:** 2022-06-17

**Authors:** Nisha Shrestha, Ann Hye-Ryong Shim, Mohammad Mehdi Maneshi, Priscilla See-Wai Yeung, Megumi Yamashita, Murali Prakriya

**Affiliations:** Department of Pharmacology, Northwestern University Feinberg School of Medicine, Chicago, Illinois, USA

**Keywords:** STIM1, Orai1, CRAC channels, calcium signaling, store-operated calcium entry, CAD, CRAC-activation domain, CC1, coiled-coil 1, CRAC, Ca2+-release activated Ca2+, ER, endoplasmic reticulum, GOF, gain-of-function, LOF, loss-of-function, OASF, ORAI1 activating small fragment, SOAR, STIM1-Orai1 activating region, TEA-Cl, tetraethylammonium chloride, TIRF, total internal reflection

## Abstract

Stromal interaction molecule 1 (STIM1) is a widely expressed protein that functions as the endoplasmic reticulum (ER) Ca^2+^ sensor and activator of Orai1 channels. In resting cells with replete Ca^2+^ stores, an inhibitory clamp formed by the coiled-coil 1 (CC1) domain interacting with the CRAC-activation domain (CAD) of STIM1 helps keep STIM1 in a quiescent state. Following depletion of ER Ca^2+^ stores, the brake is released, allowing CAD to extend away from the ER membrane and enabling it to activate Orai1 channels. However, the molecular determinants of CC1–CAD interactions that enforce the inhibitory clamp are incompletely understood. Here, we performed Ala mutagenesis in conjunction with live-cell FRET analysis to examine residues in CC1 and CAD that regulate the inhibitory clamp. Our results indicate that in addition to previously identified hotspots in CC1⍺1 and CC3, several hydrophobic residues in CC2 and the apex region of CAD are critical for CC1–CAD interactions. Mutations in these residues loosen the CC1-CAD inhibitory clamp to release CAD from CC1 in cells with replete Ca^2+^ stores. By contrast, altering the hydrophobic residues L265 and L273 strengthens the clamp to prevent STIM1 activation. Inclusion of the inactivation domain of STIM1 helps stabilize CC1–CAD interaction in several mutants to prevent spontaneous STIM1 activation. In addition, R426C, a human disease–linked mutation in CC3, affects the clamp but also impairs Orai1 binding to inhibit CRAC channel activation. These results identify the CC2, apex, and inactivation domain regions of STIM1 as important determinants of STIM1 activation.

In most animal cells, Ca^2+^-release–activated Ca^2+^ (CRAC) channels are a major route of Ca^2+^ entry and mediate a process known as store-operated Ca^2+^ entry. Activated by the depletion of endoplasmic reticulum (ER) Ca^2+^ stores (1), prototypic CRAC channels are assembled from two proteins: Orai1, the pore-forming subunit, and STIM1, the ER Ca^2+^ sensor and CRAC channel activator (1). CRAC channels regulate a wide range of functions including gene expression, immune cell function and development, astrocyte function, and neuronal signaling (2, 3, 4, 5, 6). The importance of CRAC channels for human health is underscored by genetic studies showing that patients with loss-of-function (LOF) or gain-of-function (GOF) mutations in STIM1 or Orai1 suffer from life-threatening immunodeficiencies, autoimmunity, bleeding defects, and muscle weakness (1, 7, 8). Based on these and related functions, CRAC channels have emerged as potential targets for treatment of chronic inflammation, autoimmunity, and related diseases.

Activation of CRAC channels occurs through interaction of the ER Ca^2+^ sensor, STIM1, with the cytoplasmic C-terminus of Orai1, resulting in both molecules gathering in closely apposed sites in the ER and plasma membranes (1, 9, 10, 11, 12, 13). In cells with replete Ca^2+^ stores, the C-terminus of STIM1 is maintained in a folded, inactive conformation (1, 10, 14). However, depletion of ER Ca^2+^ stores *via* activation of IP_3_ or ryanodine receptors results in dissociation of Ca^2+^ from the EF-hands in the ER lumen of STIM1, thereby triggering a conformation change that is propagated across the ER membrane into the C-terminal cytoplasmic domains of STIM1 (1, 14). This conformational change exposes an Orai1-binding domain (known as the CRAC activation domain (CAD) or the STIM1-Orai1 activating region (SOAR)) in the cytoplasmic region of STIM1 (Fig. 1*A*) that binds to the intracellular tails of Orai1 to activate CRAC channels (1, 14, 15, 16).

**Figure 1:**
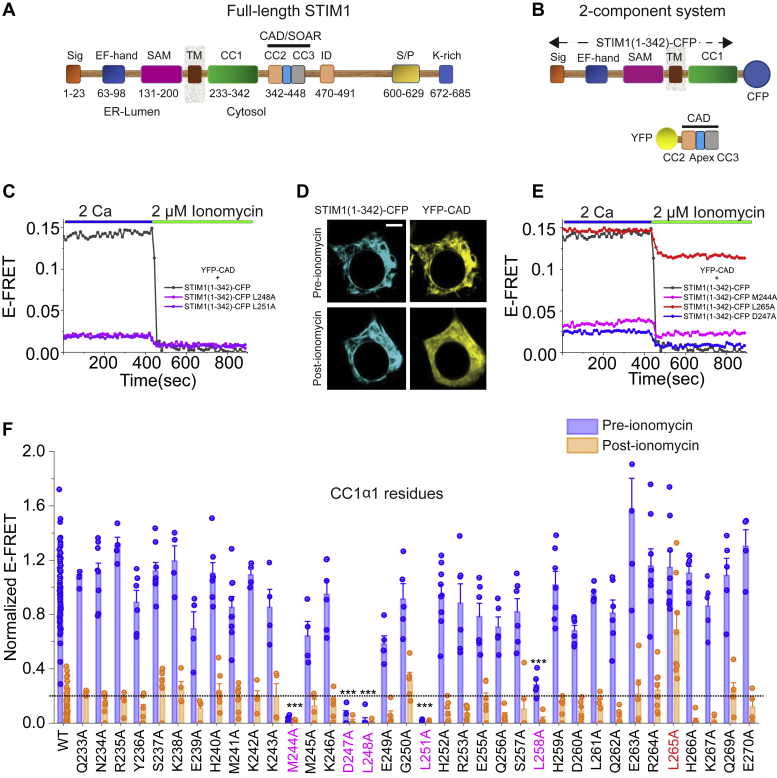
**Analysis of CC1-CAD binding and the role of CC1⍺1 residues.***A* and *B*, schematics of STIM1 and the STIM1 domains used in the FRET assay. Panel *A* illustrates the key domains in full-length STIM1. Panel *B* shows the 2-component system used in the Ala scanning screen. STIM1_1–342_ was labeled with CFP and CAD was labeled at the N-terminus with YFP. *C*, representative E-FRET time course traces of L248A and L251A STIM1 mutants which were previously shown to dissociate the intramolecular STIM1 clamp in resting cells (21, 23). In WT STIM1_1–342_ (*black trace*), E-FRET is relatively high in the resting state of the cell. Following store depletion with 2 μM ionomycin administered in Ca^2+^-free Ringer’s solution (0 mM Ca^2+^ + 1 mM EGTA), E-FRET between STIM1_1–342_-CAD rapidly declines due to dissociation of the two components. *D*, confocal images of STIM_1–342_-CFP and YFP-CAD in the resting state and following store depletion. The scale bar represents 5 μm. *E*, example E-FRET traces of the Ala mutants M244A and D247A which destabilize the intramolecular STIM1 clamp to dissociate CAD from STIM1_1–342_. By contrast, L265A strengthens the clamp such that YFP-CAD is only partially released from STIM1_1–342_ following store depletion. *F*, normalized E-FRET values of CC1⍺1 Ala mutants (Mean ± SEM; n ≥ 4) in the resting state (pre-ionomycin) and in the store-depleted state (post-ionomycin). Mutants that released the intramolecular clamp are labeled in *pink*. L265A which stabilizes the clamp is labeled in *red*. E-FRET of different mutants was normalized to the E-FRET value of WT CC1–CAD interaction (E-FRET = 0.14) at rest. Each bar represents the mean ± SEM (n = 4–8 cells for each mutant and n = 65 cells for WT). Statistical analysis was performed using one-way ANOVA followed by posthoc Dunnett test to compare pre-ionomycin values of the different mutants. One-way ANOVA: F = 403.2, *p* = 2.7e-4. Posthoc Dunnett test: ∗∗∗*p* < 0.001. The *dotted line* represents the E-FRET value of the WT construct in the store-depleted state + 2 × SD of the WT dataset. The data in *F* were collected from 3 to 5 transfections/mutant. WT data were collected from 36 transfections. CAD, CRAC activation domain; CC1, CC2, and CC3, coiled-coil domains; EF, EF-hand motif; ID, inactivation domain; K-rich, polybasic domain; S/P, proline-serine-threonine-rich segment; SAM, sterile α motif; Sig, signal peptide; TM, transmembrane segment.

A question of significant interest to the field is the molecular basis of STIM1 activation following ER Ca^2+^ store depletion. Structural models indicate that CAD, which is formed by two coiled-coil domains, CC2 and CC3, that are connected by the apex domain, assembles as a dimer with the CC2 and CC3 domains forming a hair-pin motif (17). The monomers are oriented in a roughly parallel configuration, with the N- and C-termini of each monomer facing the same direction (17). The pioneering studies of Romanin, Hogan, Zhou, Lewis, and other groups have revealed that in cells with replete stores, CAD is sequestered within a folded state of the STIM1 C-terminus *via* direct interactions with the CC1 coiled-coil domain that is adjacent to the ER membrane, to keep STIM in an inactive state (18, 19, 20, 21, 22, 23). In this state, CC1, and in particular, its first sub-domain termed CC1⍺1, functions as an “inhibitory brake” through interactions with CAD, causing the entire cytoplasmic region of STIM1 to adopt a compact and folded form (18, 19, 20). However, store depletion triggers dimerization of the luminal EF-SAM domains and releases the inhibitory clamp by packing the CC1 domains together to force CAD outward and away from the ER membrane (9, 10, 24). This extended form of STIM1 allows CAD to reach out and interact with Orai channels in the plasma membrane.

The structural details of the interaction between CC1 and CAD controlling release of the intramolecular inhibitory clamp have been the focus of much investigation but not yet fully understood. Mutagenesis and structure-function studies have implicated CC1⍺1 as a key site within the CC1 region that interacts with CAD, and several residues including L248, L251, and L258 are known to stabilize the inactive structure (19, 20, 21). Within CAD, the C-terminal region of CC3, including residues L416 and V419 have been implicated in CC1 binding (18, 19). However, a key limitation of CC1-CAD binding models developed from these studies is that they are based largely on engineered mutations of preselected residues in STIM1 fragments. Whether other regions outside of CC3 in CAD/SOAR are also involved in interactions with CC1 has never been tested. Another limitation is that the studies were largely carried out using soluble STIM1 domains lacking the luminal ER and transmembrane domains, but whether other regions of STIM1 also regulate the clamp in the full-length protein is unknown. A recent smFRET study of ctSTIM1 (the STIM1 C-terminus from the transmembrane domain to its C-terminal end) immobilized to a glass surface has reaffirmed potential interactions of CC1⍺1 with CC3 (25). However, these and other studies have been agnostic on the potential contributions of the other CAD domains including CC2 and the apex for controlling the inhibitory clamp. In addition, whether CC1–CAD interactions are regulated by domains outside of CAD and CC1 is also not well understood. What is needed is an unbiased analysis with the potential to reveal all the relevant loci in CAD that are involved in the inhibitory clamp, but this has not yet been undertaken.

In this study, we addressed these questions using Ala mutagenesis and a FRET-based live-cell assay to probe the interaction of CC1, expressed with the luminal and transmembrane domains intact (in a STIM1_1–342_ fragment), and CAD expressed separately. Our results reaffirm key roles of the CC1⍺1 and CC3 domains in the maintenance of the inhibitory clamp but also reveal essential and previously unknown roles for the CC2 and the apex domains of CAD in regulating CC1–CAD interactions. Moreover, we find that the inactivation domain (ID) of STIM1 that is located distal to CAD also modulates the inhibitory clamp. Together, these results expand our understanding of the molecular basis and regulation of the CC1-CAD inhibitory clamp.

## Results

### CC1α1 residues involved in the STIM1 inhibitory clamp

To study the molecular underpinnings of the intramolecular clamp in STIM1, we employed a FRET-based two-component system previously developed by Ma *et al.* for investigating the interaction of CC1 with CAD (19, 26). In this approach, a CFP-tagged STIM1_1–342_ domain containing the luminal, transmembrane, and CC1 regions is coexpressed together with YFP-tagged human CAD in HEK cells (Fig. 1*B*). In the resting state, tight interaction of CAD with CC1 results in elevated FRET between STIM1_1–342_-CFP and YFP-CAD and colocalization of the two proteins along tubules of the ER (Fig. 1, *C* and *D*). When stores are rapidly depleted with 2 μM ionomycin, the association of CAD with STIM1_1–342_ is disrupted, lowering E-FRET between STIM1_1–342_-CFP and YFP-CAD (Fig. 1*C*) and causing CAD to disperse from ER membrane into the cytosol (Fig. 1*D*). This “hold-and-release” method offers a way to examine in real-time the unbinding of CAD from the CC1 domain in live cells.

To determine the residues that are involved in maintaining the STIM1 intramolecular clamp, we used this two-component system together with alanine scanning mutagenesis in the CC1, CC2, and CC3 domains. We first scanned the CC1α1 region as this region has been implicated in maintaining STIM1 in the resting state in cells with replete ER Ca^2+^ stores. Previous studies using the soluble STIM1 fragments, STIM1ct and OASF (ORAI1 activating small fragment), which lack the luminal N-terminal and transmembrane domains, have shown that residues L248, L251, and L258 in CC1α1 are critical for the clamp (20, 21). Ala mutations of these residues altered the configuration of STIM1 from a folded state into an extended conformation thereby releasing CAD from CC1 (20, 21) (Fig. 1*C*). To address the potential involvement of other residues in this region, we substituted the non-alanine residues in CC1α1 to alanine and employed the two-component FRET assay to identify the residues that could play role in maintaining the clamp.

This analysis revealed that Ala substitutions at several positions including M244, D247, L248, L251, and L258 markedly diminished resting state FRET between STIM1_1–342_-CFP and YFP-CAD (Fig. 1, *C*–*F*). Moreover, the pattern of these hits, well separated by stretches of less reactive amino acids, is consistent with several turns of the coiled-coil secondary structure of CC1. As noted above, mutations at L248, L251, and L258 are known to destabilize the intramolecular STIM1 clamp and release CAD to cause constitutive activation of STIM1 (21, 23). The identification of M244 and D247 (Fig. 1, *E* and *F*) is in line with two recent studies indicating that these residues also play a key role in CC1–CAD interaction (23, 27). However, in contrast to the finding reported by Ma *et al.* (19), we did not find any effect of mutating L261, a residue that has been postulated to be located at the CAD dimer-binding interface (20). This result is, however, consistent with the findings by Muik *et al.* (21), who found only modest changes in FRET in their OASF sensors with a L261S mutation. We postulate that this difference may be related to a smaller STIM1 fragment (STIM1_1–310_) used by Ma *et al.* (19), which excluded the CC1α3 domain. Replacement of leucine with glycine in that study may also elicit additional effects not observed with the Ala substitution employed in our study.

In contrast to the release of the clamp seen at the above mutants, one locus, L265, showed the converse phenotype. At this locus, substitution of Leu with Ala diminished the release of CAD from CC1 following store depletion, such that FRET between STIM1_1–342_ and YFP-CAD remained elevated even after ionomycin administration (Fig. 1, *E* and *F*). Thus, L265A evidently stabilizes the inhibitory clamp resulting in elevated CAD-CC1 binding even following store depletion.

### The role of CC2 and CC3 residues of CAD for the intramolecular clamp

**Figure 2:**
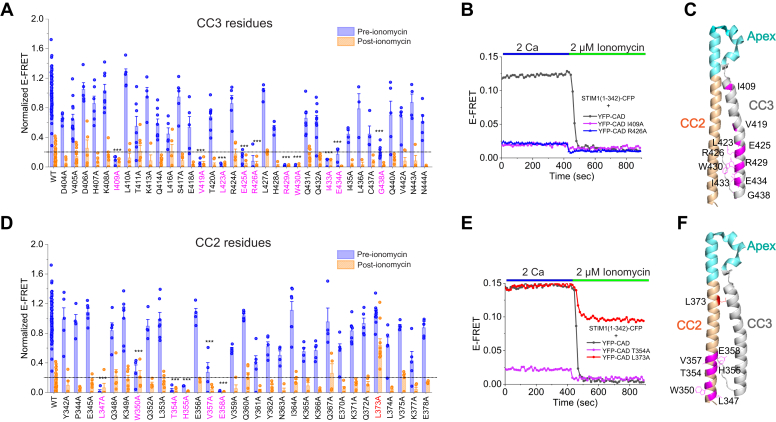
**Alanine scanof CC2 and CC3 reveals a critical role for CC2 in maintaining the intramolecular STIM1 clamp.***A*, alanine scanning of CC3 region. Residues that destabilize the CC1–CAD interaction are labeled in *pink*. The graph shows normalized E-FRET values of CC3 Ala mutants in the resting state (pre-ionomycin, *blue*) and in the store-depleted state (post-ionomycin, *orange*). Statistical analysis was performed using one-way ANOVA to compare pre-ionomycin values of the variants followed by posthoc Dunnett test. One-way ANOVA: F = 129.5, *p* = 0.007. Posthoc Dunnet test: ∗∗∗*p* < 0.001. *B*, representative time-course of CC2 hits that dissociate the clamp in quiescent state. *C*, a side-view of the monomeric crystal structure of CAD with the CC3 mutants that dissociated CAD from STIM1_1–342_ in the screen shown in *pink*. *D*, alanine scan of CC2 region with residues destabilizing CC1–CAD interaction labeled in *pink*. The residue L373A in CC2 (shown in *red*) only partially released the CAD from CC1 after store-depletion. Statistical analysis was performed using one-way ANOVA to compare pre-ionomycin values of the variants followed by posthoc Dunnett test. One-way ANOVA: F = 570.2, *p* = 1.6e-4. Posthoc Dunnet test: ∗∗∗*p* < 0.001. *E*, representative time-course of hit mutants that dissociate the clamp in resting state (*black*), T354A (*pink*), and L373A (*red*) which affect the clamp. *F*, a side-view of the monomeric crystal structure of CAD with the CC2 mutants disrupting CAD-CC1 association shown in *pink*. Each *bar* in panels (*A*) and (*D*) is the mean ± SEM (4–10 cells for each mutant and n = 65 cells for WT condition). The *dotted line* indicates the WT E-FRET value in the store-depleted state + 2 × SD. The data in (*A*) and (*D*) were collected from 3 to 6 transfections/mutant. WT data were collected from 36 transfections. CAD, CRAC-activation domain; CC1, coiled-coil 1.

In the prevailing model of STIM1 activation, CC1α1 is thought to interact with CC3 of CAD to maintain STIM1 in a folded state at rest (18, 19). Consistent with this model, our scan of CC3 identified several residues including I409, V419, L423, E425, R426, R429, W430, I433, E434, and G438 whose mutation to Ala released the CC1-CAD clamp (Fig. 2, *A* and *B*). Residues V419 and L423 have been previously implicated in the interaction of CC1 with CAD/SOAR (19), which is in line with our findings. A conservative Ala substitution of L416 also reduced CC1-CAD E-FRET more moderately, consistent with previous reports indicating that nonconservative substitutions of L416 (L416G and L416S) activate STIM1 (19, 21). Together, these results confirm previous findings implicating the distal C-terminal region of the CC3 domain for CC1 binding and also identify new residues (I409, E425, R426A, I433) that are involved in this interaction (Fig. 2*C*).

Because prevailing models and previous studies have not identified a role for CC2 in STIM1 activation, we expected that Ala mutations in CC2 would not affect CC2-CAD binding. Yet, surprisingly, a scan of CC2 (a.a. 342–378) revealed a large cluster of residues in CC2, including L347, W350, T354, H355, V357, and E358 that regulated CC1-CAD binding. Ala mutations of these residues strongly diminished FRET between STIM1_1–342_-CFP and YFP-CAD in resting cells (Fig. 2, *D* and *E*). Notably, these residues were located predominantly toward the base of the CC2 segment on consecutive turns of the CC2 helix (Fig. 2*F*). By contrast, Ala mutations in the central and C-terminal region of CC2 did not release the intramolecular clamp. L347 and W350 are implicated in hydrophobic interactions with residues L436 and I433 in CC3 within the same monomer (17), raising the possibility that the Ala substitutions may disrupt dimerization of CAD. However, tests using CAD-CAD FRET to probe CAD dimerization showed that single Ala mutations of L347 and W350 did not diminish CAD dimerization (E-FRET between YFP-CAD/CFP-CAD for WT CAD: 0.11 ± 0.006; n = 58 *versus* 0.13 ± 0.006, n = 48 for L347A and 0.11 ± 0.009, n = 42 for W350A). Taken together, these results indicate that the CC2 domain also significantly contributes to CC1–CAD interaction.

In contrast to the Ala hits described above that disrupted CC1-CAD binding, we observed that an Ala mutation at L373 strengthened CC1–CAD interaction (Fig. 2, *D* and *E*). In resting cells, L373A YFP-CAD showed comparable FRET with STIM1_1–342_-CFP. After store depletion, however, FRET between these interacting partners remained elevated and decreased by only a fraction of the change seen in WT CAD (Fig. 2, *D* and *E*). Thus, as seen earlier for the CC1α1 L265A mutant (Fig. 1*E*), L373 appears to be an important locus for tuning the affinity of CC1–CAD interaction to permit CAD to disengage from CC1 upon store depletion. The molecular basis of this phenotype is described further below but first we investigated the apex resides.

### The apex domain of CAD regulates CC1-CAD binding

**Figure 3:**
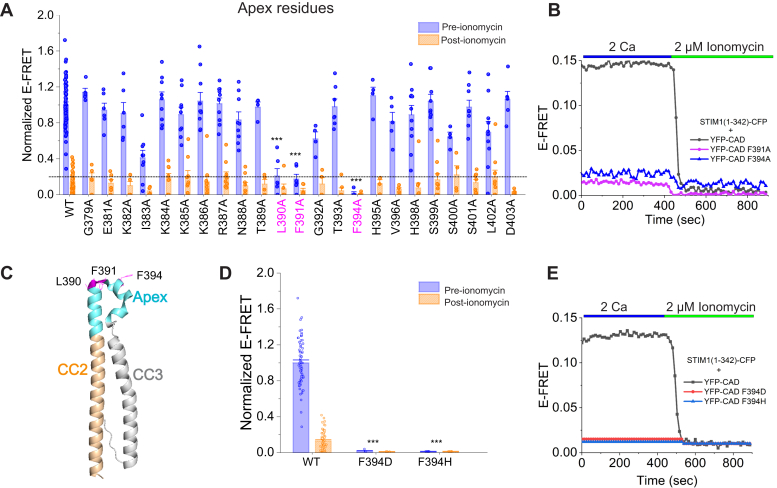
**Alanine scanofthe CADapex regionindicates key roles for some apexresiduesin stabilizing the intramolecular clamp.***A*, normalized E-FRET values of the CAD apex Ala mutants in the resting state (pre-ionomycin, *blue*) and in the store-depleted state (post-ionomycin, *orange*). Residues L390, F391, and F394 (highlighted in *pink*) destabilize the CC1–CAD interaction in quiescent state. *B*, representative time-course traces of F391A (*purple*) and F394A (*blue*) before and following exposure to 2 μM ionomycin. (Mean ± SEM; n ≥ 4). *C*, a side-view of the monomeric crystal structure of CAD with the apex mutations disrupting CAD-CC1 association represented in *pink*. *D*, summary of the normalized E-FRET values of WT and F394D and F394H mutants. F394D and F394H completely disrupt the inhibitory clamp. *E*, representative time-courses of E-FRET changes between STIM1(1–342)-CFP and YFP-CAD (F394D and F394H). Each *bar* in (*A*) and (*D*) represents the Mean ± SEM of 4 to 11 cells for each mutant and n = 65 cells for WT. Statistical analysis was performed using one-way ANOVA to compare pre-ionomycin values of the variants followed by posthoc Dunnett test. One-way ANOVA: F = 1933, *p* = 2.5e-5. Posthoc Dunnett test: ∗∗∗*p* < 0.001. The data in (*A*) were collected from 3 to 4 transfections/mutant. WT data were collected from 36 transfections. CAD, CRAC-activation domain; CC1, coiled-coil 1.

The “apex” region of the CAD/SOAR domain, which links CC2 and the CC3 domains through two short helices, has been implicated in Orai1 binding and channel gating in several studies (28, 29, 30). However, it is unknown if this region also plays a role in regulating the CC1-CAD inhibitory clamp. To address this question, we next scanned all the residues in the CAD apex region. This scan showed that Ala mutation of three apex residues, L390, F391, and F394 in the apical ⍺3 helix disrupted the intramolecular clamp and released CAD from CC1 (Fig. 3, *A*–*C*). F394 has been extensively studied and previous reports indicate that several mutations at F394 including F394D and F394H impair STIM1-Orai1 binding and CRAC channel gating (30). We observed that, as seen for F394A, both F394H and F394D also disrupted CC1-CAD binding and released YFP-CAD from the ER to reduce E-FRET with STIM1_1–342_-CFP, with F394D eliciting even lower levels of CC1 binding at rest as assessed by E-FRET than F394A (Fig. 3, *D* and *E*). Likewise, individual Ala mutations of L390 and F391 released CAD from CC1 in resting cells (Fig. 3*A*). L390 and F391 are implicated in Ca^2+^-calmodulin binding and a double L390S/F391S mutation was previously found to impair Orai1 binding and channel activation (31). Thus, the ability of Ala mutations of L390, F391, and F394 in the apex to interfere with CC1 binding suggests that these hydrophobic residues have multiple roles in controlling not only Orai1 binding and gating, but also STIM1 activation.

### L265 and L373 regulate the stability of the CC1-CAD clamp

**Figure 4:**
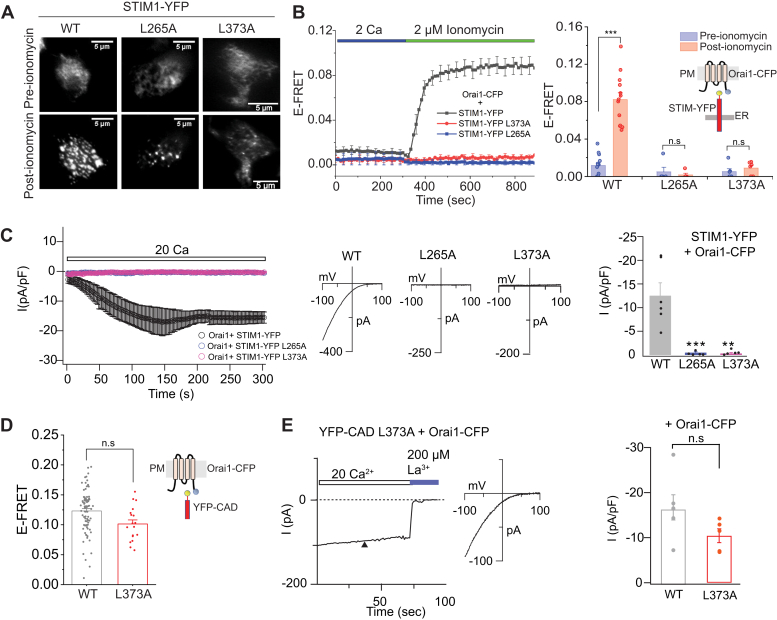
**The L265A and L373A mutations prevent STIM1 activation.***A*, TIRF images of STIM1-YFP L265A and L373A at rest and following store depletion. Images were collected in resting cells in 2 mM extracellular Ca^2+^ and 3 min after store depletion by 2 μM ionomycin in Ca^2+^-free solution. Please note that the WT images were collected together with the data in Figure 6*A* and also appear in Figure 6*A*. *B*, time course of E-FRET between L373A and L265A STIM1-YFP and Orai1-CFP following ionomycin administration. Whereas WT STIM1-YFP shows robust increases in E-FRET with Orai1-CFP following store depletion, L373A and L265A STIM1-YFP are impaired in Orai1 binding. *Right panel*, summary of the E-FRET changes between STIM1-YFP and Orai1-CFP in the indicated mutants in the FRET experiments shown in (*B*). *C*, time course of the I_CRAC_ induction by WT or mutant STIM1-YFP in cells coexpressing Orai1-CFP. I_CRAC_ was induced by passive depletion of ER Ca^2+^ stores by 8 mM BAPTA in the internal recording solution. The *middle plot* shows the loss-of-function phenotype of L373A and L265A STIM1 mutants. The current-voltage relationships of the indicated mutants are shown on the *right*. The *bar graph* on the *right* summarizes the current amplitudes of the indicated mutants at t = 300 s ∗∗∗*p* < 0.001 by unpaired *t*-tests between the pre- and post-ionomycin conditions for each mutant. Please note that the WT current traces also appear in Fig. S1*C*. *D*, E-FRET between YFP-CAD and Orai1-CFP is not affected by the L373A CAD mutation. This result indicates that L373A does not directly impair STIM1 binding to Orai1. Each bar is the mean ± SEM of ≥4 cells. ∗∗∗*p* = 0.00028. F = 16.27 by one-way ANOVA followed by posthoc Tukey test. *E*, I_CRAC_ is activated normally by the L373A CAD mutant. The *left plot* shows the time course of I_CRAC_ activation in a cell expressing L373A CAD with Orai1-CFP. The *middle panel* shows the current-voltage relationship and the *right bar graph* summarizes the current amplitude in cells expressing either WT or L373A CAD with Orai1-CFP. CAD, CRAC-activation domain; CRAC, Ca^2+^-release activated Ca^2+^; ER, endoplasmic reticulum; TIRF, total internal reflection.

As shown in Figures 1 and 2, L265A in CC1⍺1 and L373A in CAD stabilize CC1-CAD binding such that store depletion fails to fully uncouple CAD from CC1 in these mutants. To examine the consequences of this stabilization for STIM1 activation, we introduced these mutations into full-length STIM1 and assessed STIM1 activation by monitoring puncta formation following store depletion. Consistent with the observation that L265A and L373A stabilize CC1⍺1–CAD interactions, STIM1-YFP puncta formation was impaired in both mutants (Fig. 4*A*). Further, FRET measurements between STIM1-YFP and Orai1-CFP indicated that the interaction of L265A STIM1 and L373A STIM1 with Orai1 was also markedly reduced following store depletion (Fig. 4*B*). Consistent with these LOF effects, measurements of CRAC currents by patch-clamp electrophysiology revealed that Orai1 currents arising from L265A and L373A STIM1 were significantly reduced compared to WT STIM1 (Fig. 4*C*). Interestingly, introduction of L373A into CAD revealed no defect in its interaction with Orai1 as assessed by FRET (Fig. 4*D*). Thus, the LOF phenotype of L373A STIM1 is not due to intrinsic defect in the binding of CAD to Orai1 but appears entirely due to the ability of the mutation to prevent STIM1 activation following store depletion. In line with this result, no defect was observed in L373A CAD-mediated activation of Orai1 currents (Fig. 4*E*) indicating that the mutation does not inherently affect the ability of CAD to gate Orai1 channels. Together, these results indicate that L265A and L373A stabilize the interaction of CAD with CC1 to block release of the intramolecular STIM1 clamp. The finding that L373A impairs STIM1 activation of Orai1 channels is interesting as a previous study found that a nonconservative substitution of this locus (L373S) inhibits STIM1 coupling to Orai1 and CRAC current (32), which was interpreted to arise from defective STIM1-Orai1 binding. Our results suggest that the defect is likely due to impaired STIM1 activation rather than Orai1 binding.

### The size of the side-chain at L373 is an important determinant of CAD-CC1 binding

**Figure 5:**
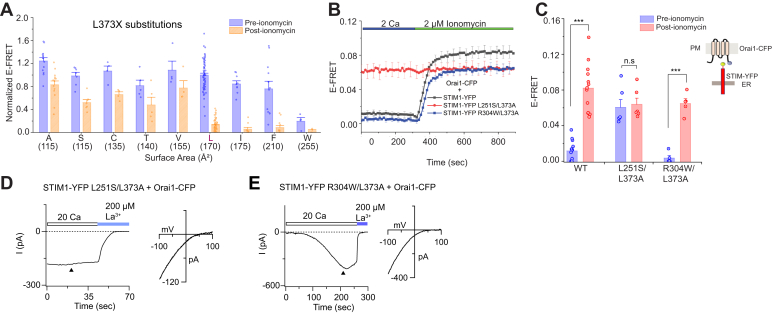
**The size of the side-chain at L373 regulates the stability of the intramolecular clamp.***A*, replacement of L373 with residues smaller than the native leucine partially release the clamp upon store depletion in two-component assay, whereas insertion of amino acids larger in size than leucine at this position (labeled in *pink*) disrupt CC1 binding in the resting state. The *bar graph* summarizes the E-FRET between the indicated YFP-CAD mutants and STIM1_1–342_-CFP using the two-component assay. *B*, introduction of the constitutively active disease mutation, R304W, in full-length STIM1 L373A restores STIM1 activation following store-depletion. E-FRET is low in resting cells and comparable to the FRET seen with WT STIM1-YFP but increases following store depletion. In contrast, the GOF mutation L251S, causes constitutive activation of L373A STIM1. *C*, summary of the E-FRET data shown for the different mutants in *B*. Mean ± SEM, n ≥ 4 cells per mutant, ∗∗∗*p* < 0.0001 by unpaired *t* test. *D* and *E*, example CRAC currents recordings in cells expressing Orai1-CFP and L251S/L373A STIM1 (*D*) or R304W/L373A STIM1 (*E*). Cells expressing L251S/L373A STIM1 show large standing inward currents at whole-cell break-in (at t = 0 s). By contrast, R304W/L373A STIM1 shows a store-operated Orai1 current that slowly activates following whole-cell break-in. CAD, CRAC-activation domain; CC1, coiled-coil 1; CRAC, Ca^2+^-release activated Ca^2+^; GOF, gain-of-function.

To further investigate the molecular basis of the L373A phenotype, we carried out extended mutagenesis of the native Leu at this position. At rest, E-FRET between STIM1_1–342_-CFP and YFP-CAD was largely unaffected by different mutations at this position with the notable exception of a Trp substitution which dissociated the CC1–CAD complex (Fig. 5*A*). However, following store depletion, we found that reducing the size of the side-chain at Leu 373 to the small amino acids Ala, Ser, Cys, Thr, and Val caused stronger interaction of CAD with CC1 following ER Ca^2+^ store store-depletion than that seen with WT CAD (Fig. 5*A*). By contrast, the larger side-chains of the Ile and Phe substitutions showed WT-like behavior, with E-FRET between STIM1_1–342_-CFP and YFP-CAD declining fully following store depletion (Fig. 5*A*). Thus, the amino acid side-chain size at position 373 is an important determinant of the stability of CC1–CAD interaction in the store-depleted state of the ER, with smaller amino acids strengthening the interaction to prevent the release of the intramolecular clamp. We postulate that large size of the native Leu (∼170 Å^2^) at this position ensures that the affinity of CAD-CC1 binding is not too tight, thereby allowing dissociation of the complex following store depletion.

To further analyze whether the LOF phenotype of the L373A STIM1 is due to stabilization of the intramolecular clamp or another aspect of STIM1 activation or Orai1 binding, we assessed the effects of introducing two GOF mutations, L251S and R304W on the behavior of L373A STIM1. L251S is a potent GOF mutation that activates STIM1 and store-operated Ca^2+^ entry by disrupting the interaction of CC1⍺1 with CAD (20, 21). L251S induces packing of the CC1 domains to extend CAD away from CC1, thereby permitting CAD to interact with Orai1 and activate CRAC channels constitutively in the absence of store depletion (23). By contrast, R304W, a naturally occurring human mutation linked to tubular aggregate myopathy (33), is not thought to be located directly at the CC1-CAD binding interface. Instead, Trp mutation of this residue is postulated to destabilize packing of the CC1⍺3 domains against each other in the resting state to allosterically uncouple CC1⍺1 from CAD and extend the CC1 domains away to activate STIM1 (34). We introduced these GOF mutations into L373A STIM1-YFP and examined the ensuing effects on STIM1–Orai1 interaction at rest and following store depletion with 2 μM ionomycin (Fig. 5*B*).

These experiments showed that the addition of L251S easily overcame the LOF phenotype of the L373A mutation. L251S/L373A STIM1-YFP showed strong constitutive interaction with Orai1-CFP (Fig. 5, *B* and *C*), analogous to the phenotype of the single L251S mutation which activates STIM1. Evidently, the GOF mechanism of L251S dominates the LOF phenotype of L373A. By contrast, R304W showed the converse phenotype—in this mutant, the constitutive activity of R304W was lost, and the R304W/L373A double STIM1 mutant exhibited little, if any, constitutive Orai1 binding (Fig. 5, *B* and *C*). However, following store depletion, the R304W/L373A STIM1 showed robust Orai1 binding comparable to the behavior of WT Orai1 indicating that in this mutant, the effects of L373A dominates the allosteric GOF phenotype of R304W. In line with these STIM1-Orai1 FRET phenotypes, electrophysiological recordings indicated that cells expressing L251S/L373A STIM1 showed large standing inward CRAC currents visible immediately following whole-cell break-in (Fig. 5*D*), indicating that the constitutively active L251S/L373A STIM1 mutant tonically activates Orai1. By contrast, CRAC currents in cells expressing Orai1+ R304W/L373A STIM1 were activated only following store depletion with dialysis of BAPTA into the cell (Fig. 5*E*). Together, these results indicate that unlike the L251S mutation, R304W is unable to overcome the activation barrier of the L373A STIM1 mutation. These opposing phenotypes indicate that L373A can differentiate between the differing underlying mechanisms of R304W and L251S in activating STIM1.

### STIM1 ID promotes CC1–CAD interaction

**Figure 6:**
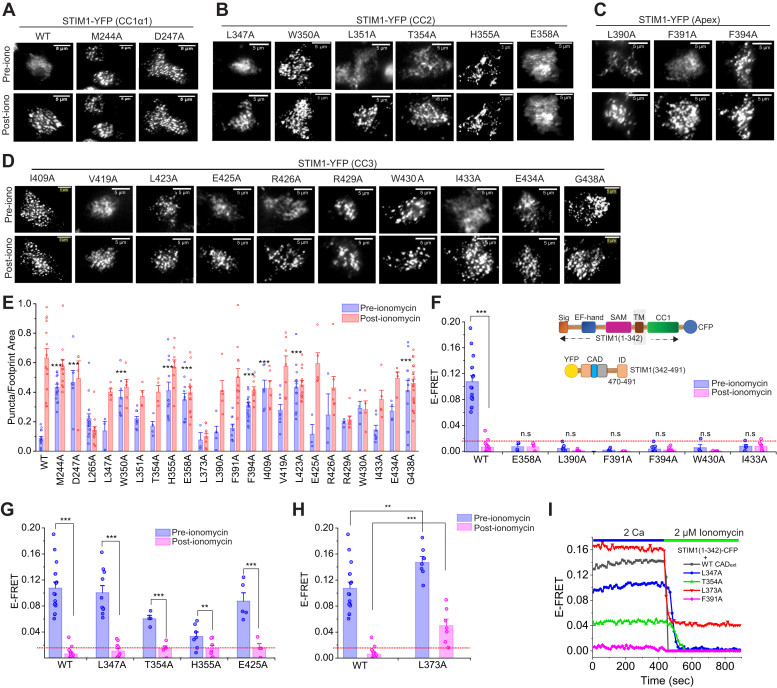
**The inactivation domain (ID) of STIM1 regulates CC1–CAD interaction.***A*–*D*, TIRF images of the “hit” mutants identified from alanine scanning (Figure 1, Figure 2, Figure 3) introduced into full length STIM1 in the resting state and following store depletion with 2 μM ionomycin. Several mutations including M244A, D247A in CC1⍺1 and W350A, F391A, F394A, L423A, and W430A form punctae to varying degrees in resting cells prior to ionomycin treatment (the scale bar represents 5 μm). Please note that the WT column images in (*A*) are the same as those shown in the WT column images of Figure 4*A*. *E*, quantification of STIM1-YFP puncta formation in resting cells and following store depletion with 2 μM ionomycin using particle analysis. ∗∗∗*p* < 0.001 by one-way ANOVA to compare the pre-ionomycin puncta values (F = 7.66, *p* = 1.1e-16) followed by posthoc Tukey test. *F*, a schematic representation of the constructs STIM1_1–342_-CFP and YFP-CAD_ext_ (STIM1_342–491_). The *bar graph* shows E-FRET of STIM1_1–342_-CFP with the indicated CAD_ext_ mutants. These mutants show low FRET at rest indicating that the mutations dissociate the CC1–CAD complex. ∗∗∗*p* < 0.001 by unpaired *t* test. *G*, by contrast, L347A, T354A, and E425A show elevated E-FRET indicating strong CC1 binding with CAD_ext_ in resting cells. *p* < 0.001 (∗∗∗) or *p* < 0.01 (∗∗) by unpaired *t* test comparing pre- and post-ionomycin values in each mutant. *H*, resting E-FRET for L373A is higher than the WT CAD_ext_. Moreover, store depletion fails to fully release CAD_ex__t_ from CC1 in this mutant indicating incomplete dissociation of the CC1-CAD complex (mean ± SEM, n ≥ 5 cells per mutant). *p* < 0.001 (∗∗∗) or *p* < 0.01 (∗∗) by unpaired *t* test comparing pre- and post-ionomycin values in each mutant. *I*, example time course traces showing E-FRET changes between STIM1_1–342_-CFP and the indicated YFP-CAD_ext_ constructs at rest and following store depletion. CAD, CRAC-activation domain; CC1, coiled-coil 1; TIRF, total internal reflection.

To examine whether the novel hits identified in our CC1-CAD binding screen in CC1⍺1 and CAD also cause constitutive activation of full-length STIM1, we next introduced these mutations into STIM1-YFP and examined the degree of puncta formation of STIM1-YFP using total internal reflection (TIRF) microscopy. This analysis showed that in CC1⍺1, STIM1 M244A and D247A showed punctate distribution even in resting cells with replete ER Ca^2+^ stores (Fig. 6*A*). Moreover, FRET measurements showed high levels of FRET between STIM1-YFP and Orai1-CFP in the M244A and D247A STIM1 mutants in resting cells (Fig. S1, *A* and *B*), which is consistent with the constitutive release of the intramolecular clamp leading to STIM1 activation in these mutants. Electrophysiological analysis of CRAC currents in cells expressing Orai1-CFP with the mutant STIM1 proteins revealed that CRAC currents were constitutively activated in STIM1 M244A and D247A expressing cells (Fig. S1, *C* and *D*), in contrast to the LOF phenotype of STIM1 L265A described earlier (Fig. 4).

Likewise, in CAD, several hits in the STIM1_1–342_-CAD FRET screen that dissociated CAD from CC1 (Fig. 6, *B*–*D*), including W350A, H355A, F394A, I409A, L423A, R429A, W430A, E434A, and G438A all caused significant spontaneous STIM1 puncta formation as expected from release of the inhibitory clamp (Fig. 6, *B*–*E*). Thus, disrupting these CC1⍺1 and CAD residues triggers STIM1 activation independently of store depletion. Importantly, the presence of several spontaneous active CC2 and apex mutants reaffirms the role of these domains in controlling STIM1 activation by regulating the CC1-CAD intramolecular clamp.

Yet, other mutations in CAD did not spontaneously activate full-length STIM1. For example, L347A and L354A in CC2, F391A at the apex, and E425A and I433A in CC3 remained distributed in the ER (Fig. 6, *B*–*D*), despite the ability of these mutations to disrupt the CC1-CAD clamp in the two-component assay (Figs. 2 and 3). Notably, these phenotypes were conspicuously different from the phenotypes seen in the CC1α1 domain, where the mutations M244A and D247A that disrupted the CC1-CAD clamp also spontaneously caused puncta formation of full-length STIM1 (Fig. 6*A*). This result raised the possibility that additional regions in the full-length STIM1 sequence must contribute to the stability of CC1-CAD binding in cells with replete ER Ca^2+^ stores.

What might be the identity of these additional domains? It has been recently reported that the ID of STIM1 (a.a. 470–491) allosterically facilitates CC1-CAD binding, and therefore, helps maintain the inhibitory clamp in the resting state (35). We postulated that the absence of ID_STIM1_ in the CAD construct employed in the above experiments may render the clamp more sensitive to mutations that disrupt CC1 binding. To address this hypothesis, we extended the length of the CAD domain by 43 amino acids to include ID_STIM1_ (a.a. 342–491). This extended CAD domain, which we called CAD_ext_ (CAD_342–491_), was then tested in the 2-component FRET assay (Fig. 6*F*, schematic).

As shown in Figure 6*G*, several CC2 and CC3 Ala mutants that fully released CAD in the CC1-CAD FRET assay (*i.e.*, showed low resting E-FRET between CC1-CFP and YFP-CAD, Fig. 2) retained elevated levels of E-FRET in the longer CAD_ext_ fragment. Specifically, L347A, T354A, and E425A, which all caused spontaneous release of CAD (Fig. 2) showed elevated levels of CC1-CAD_ext_ binding in the resting state (Fig. 6, *G* and *I*). Following store depletion with 2 μM ionomycin, E-FRET between CC1-CFP and WT YFP-CAD_ext_ declined to levels comparable to those seen with CAD following store depletion (Fig. 6, *G* and *I*). To a smaller extent, this phenotype was also visible in H355A. CAD_ext_-L373A exhibited even higher relative E-FRET with CC1-CFP (Fig. 6*H*) compared to that seen with the shorter CAD_342–448_ (Fig. 2), indicating that L373A stabilizes CC1-CAD binding. Thus, these results indicate that extending CAD by 43 amino acids to include ID_STIM1_ stabilizes the binding of CAD to CC1 in the resting state in several spontaneous active CAD mutants. Other mutants however, including E358A, L390A, F391A, and F394A remained unbound to CC1 even in the longer CAD_ext_ domain (Fig. 6*F*) indicating that the addition of the ID_STIM_ was not enough to fully restore CAD binding to CC1 in these mutants. Nevertheless, the stabilization of CAD_ext_-CC1 binding in the resting state in the L347A, T354A, H355A, and E425A mutants indicates that the addition of ID_STIM1_ to CAD stabilizes interaction of CAD with CC1 in these mutants.

### The disease-linked R426C mutation evoked complex effects on STIM1 activation

Residue R426 is a known disease-causing locus with one case report documenting impaired dental enamel formation and nail dysplasia in a 6-years child with the R426C substitution (36). Although immunological evaluation was not performed in the patient, the apparent defect in amelogenesis is consistent with impaired STIM1 function. However, to date, no functional studies have been reported on this STIM1 mutation. Given that the R426A mutation strongly destabilized the CC1-CAD clamp in the two-component system, we therefore sought to understand the functional consequences of R426C for STIM1 activation and CRAC channel activation. We found that in the two-component assay, introduction of the R426C mutation into CAD caused dissociation of CAD from CC1 (Fig. S2*A*). This effect is similar to the phenotype seen with R426A (Fig. 2*A*), indicating that the disease mutation destabilizes the intramolecular clamp. However, because a previous study has noted that a Leu mutation at this locus (R426L) prevents STIM1 from interacting with Orai1 (21), we also examined the effects of R426C for Orai1 binding and activation. These experiments revealed that R426C moderately but significantly inhibited the interaction of CAD with Orai1 (Fig. S2*B*). To a greater extent, R426C also inhibited STIM1 interaction with Orai1 (Fig. S2*C*). Consistent with this defect in Orai1 binding, electrophysiological analysis showed that Orai1 currents activated by R426C were significantly reduced compared to WT STIM1 (Fig. S2*D*). Together, these results indicate that R426C evokes multiple effects on STIM1 function, causing both destabilization of the CC1-CAD clamp but also preventing STIM1 from effectively engaging with and activating Orai1 channels. The latter effect may explain the overall LOF phenotype of the mutant for CRAC channel activation its links to defective enamel formation and dysplasia (36).

## Discussion

STIM1 is activated by a molecular mechanism involving relief of an inhibitory clamp within the cytoplasmic region that unmasks the catalytic CAD domain. Once exposed, CAD is free to reach across the junctional space separating the ER and the plasma membranes to interact with and activate Orai channels. The molecular mechanism underlying removal of the inhibitory clamp to trigger STIM1 activation and the mechanisms controlling its regulation are major questions of interest, and insightful studies from Romanin, Hogan, Zhou, Lewis, and other groups have revealed that residues in CC1⍺1 and CC3 domains play a critical role in maintaining the inhibitory clamp (10, 22). In this study, we show that the residues in the CC2 and the apex region of CAD are also essential for the maintenance of the clamp and mutation of these residues causes spontaneous release of the inhibitory clamp in resting cells. Moreover, we show that the ID region of STIM1, which was previously implicated in controlling inactivation of CRAC channels (37), is involved in regulating the inhibitory clamp. Together, these findings identify new molecular checkpoints that control the release of the STIM1 inhibitory clamp.

Previous studies using the isolated fragment encompassing the cytoplasmic portion of STIM1 (STIM1ct) found that the fragment adopts a compact, folded form in the resting state (10, 18, 19, 20, 21, 22). Mutational analysis of CC1 or CAD provided evidence that the clamp is maintained by specific molecular interactions between CC1⍺1 and the CC3 domains of CAD, with alteration of key residues in CC1⍺1 (L248, L251, L258) or CC3 (L416, L423) causing spontaneous STIM1 activation (19, 20, 22, 23). These studies were reaffirmed by a recent study using smFRET measurements that found that CC1⍺1 is aligned along the length of the CC3 in a domain-swapped manner within the folded CC1-CAD structure (25). However, the role of the other domains in regulating the clamp is less understood.

Here, we find that in addition to residues in CC3, mutations of several residues in CC2 release the clamp and dissociate CAD from CC1. We suggest that the inhibitory clamp involves not only interactions of CC1⍺1 with CC3, but also with CC2. This conclusion is consistent with the structural model developed by van Dorp *et al.* (25), which indicated that in addition to CC3, CC2 is also aligned along the length of CC1⍺1. The smFRET measurements in this study indicate that the distances between *cis* CC2 and CC1⍺1 are in the same range as the distances between *cis* CC3 and CC1⍺1 (25), likely explaining why mutations at CC2 dissociate the CC1⍺1-CAD clamp.

We also found that mutations of several key residues in the apex region of CAD released the inhibitory clamp. These positions include L390, F391, and F394. Residue F394 is particularly notable as this locus has been implicated in functional interactions with Orai1 and several substitutions (F394A, F394D, F394H) are known to reduce Orai1 binding (30). We found that all three mutations also dissociated the clamp (Fig. 3), and when examined for the Ala mutation, this led to spontaneous STIM1 puncta formation (Fig. 6*A*). L390 and F391 are additionally implicated in slow Ca^2+^-dependent inactivation through a mechanism thought to involve calmodulin binding (31). Our finding that perturbing these residues also destabilizes the clamp raises the interesting possibility that the apex residues are involved in switching mechanism where they change their interaction from CC1 in the resting state to Orai1 in the active state. As a consequence, disrupting these residues not only affects Orai1 binding following store depletion but also releases the clamp. The finding that F394A activates STIM1 independently of store depletion is qualitatively similar to findings reported in a very recent study that was published during the preparation of our article (38). However, based on FRET measurements in the soluble OASF fragment (STIM1 a.a. 233–474), this study found that F394A/H/D mutations do not affect the intramolecular clamp (38). Although the reasons for the difference of the behavior of the F394 mutations in OASF from the two-component system and that of full-length STIM1 need to be fully addressed, it may be related to the fact that OASF is missing the transmembrane and ER luminal domains (38). The absence of these domains in OASF may produce non-native configurations of CC1-CAD domains leading to differing phenotypes from that seen in full-length STIM1.

In addition to the numerous loci that perturbed CC1-CAD binding in CC2, we found that Ala mutations of L265 and L373 strengthened the clamp such that ER Ca^2+^ store depletion fails to fully release CAD from CC1. In full-length STIM1, these mutations also prevent STIM1 activation and activation of Orai1 channels. Structure-function analysis suggested that the stabilization of L373A is mimicked by a variety of amino acid substitutions that reduce the side-chain size at this locus (L373A/S/V/C/T). A previous study that analyzed the L373S mutation concluded that the mutation prevents Orai1 binding (32). However, our results suggest that some of the previously described LOF effects of the L373S mutation on Orai1 binding and activation may arise from strengthening of the inhibitory clamp rather than abrogation of CAD-Orai1 binding.

A surprising aspect of our findings is that a handful of CAD mutations (L347A, T354A, E425A) that disrupted the clamp in the simplified STIM1_1–342_-CFP and YFP-CAD binding assay failed to spontaneously activate full-length STIM1. We postulate that this may be due to the presence of additional brakes or domains that stabilize the CC1-CAD inhibitory clamp. One such domain that has been suggested to allosterically stabilize the clamp is ID_STIM1_ (35). ID_STIM1_ contains a cluster of acidic residues that are also implicated in fast calcium-dependent inactivation of STIM1-gated Orai1 currents (37). We found that extending the CAD domain to include ID_STIM1_ (in the CAD_ext_ fragment) stabilized binding of STIM1_1–342_ to the extended CAD domain in the resting state in several CAD mutants that were spontaneously active (Fig. 6). This result suggests that ID_STIM1_ helps stabilize the CC1-CAD clamp such that mutations in CAD that diminish CC1-CAD binding are less effective when CAD includes ID. Because ID_STIM1_ is not known to directly interact with CAD (35), we postulate that this stabilization occurs through an allosteric influence rather than direct binding with the ID region.

It is also important to appreciate that the finding that the reason some “hits” found in the two-component assay did not activate full-length STIM1 could be related to a potential limitation of the two-component system in that it may not fully reflect all the steps involved in activation of full-length STIM1. Their limitations could include the possibility that the CAD apex may not be at sufficient proximity to the ER membrane (potentially explaining why the apex residue mutations at 390 and 391 do not activate full-length STIM1 even though they release CAD from CC1) and the possibility that CAD binds to CC1 in an altered manner due to increased degrees of freedom compared to full-length STIM1. It is also possible that there are domains in addition to ID that also contribute to the intramolecular brake which remain to be discovered. Perhaps critically, as STIM1 activation depends on and requires STIM1 oligomerization (39), it is also likely that the CC1-CAD dissociation and oligomerization steps are regulated independently. In this case, perturbations that relieve the CC1-CAD clamp may not necessarily activate STIM1 if oligomerization is also not triggered. These possibilities are not mutually exclusive and may together explain differences seen in some instances in the behavior of the 2-component system and full-length STIM1.

On the basis of these results, we propose a revised model of the STIM1 inhibitory clamp wherein interactions between several domains of CAD with CC1⍺1 are involved in maintaining STIM1 in the quiescent state in resting cells. In addition to the CC3 domain that was previously implicated, both the CC2 as well as the apex domains are involved in enforcing the clamp. As suggested from the recent smFRET-based model of CAD (25), some residues in CC2 likely interact with CC1⍺1 along the length of their coiled-coil helices. In the smFRET model of CC1-CAD, the apex domain of CAD is located close to the ER membrane. We propose that the residues of the apex, especially the short ⍺2 helix may be positioned sufficiently close to CC1⍺1 to engage in hydrophobic interactions with residues at the base of the CC1⍺1 domain. This interaction between CC1⍺1 and the apex domain could ensure that the catalytic region of CAD, the apex, is stably kept as far away from Orai channels in resting cells as possible, helping maintain STIM1 in a quiescent state. Although the precise molecular interactions between the CAD apex and membrane proximal regions of CC1 remain to be elucidated, the results presented here provide a framework for testing these and other hypotheses.

## Experimental procedures

### Cells

HEK293-H cells (Thermo Fisher Scientific) were maintained in suspension at 37 °C with 5% CO2 in CD293 medium supplemented with 4 mM GlutaMAX (Invitrogen). The HEK293 cell line is a permanent line established from primary embryonic human kidney and transformed with sheared human adenovirus type 5 DNA. For imaging and electrophysiology, cells were plated onto poly-L-lysine–coated coverslips 1 day before transfection and grown in a medium containing 44% Dulbecco’s modified Eagle’s medium (Corning), 44% Ham’s F12 (Corning), 10% fetal bovine serum (HyClone), 2 mM glutamine, 50 U/ml penicillin and 50 μg/ml streptomycin.

### Plasmids

The STIM1-YFP construct has been previously described (13). Human Orai-CFP cloned into pECFP-N1 vector (Clontech) was purchased from Addgene. STIM_1–342_-CFP was generated by amplifying this region *via* PCR and inserting the DNA into pCYFP-N1 vector (Clontech) to yield STIM1_1–342_-CFP. CFP-CAD was the kind gift of R. S. Lewis (Stanford University). YFP-CAD and YFP-CAD_ext_ (STIM1_342–491_) constructs were generated by amplifying the relevant regions *via* PCR and inserting the product into pEYFP-C1 vector between EcorI and BamHI restriction sites. All mutants (including for alanine scanning) were generated using QuickChange Mutagenesis kit (Agilent Technologies) and all constructs and mutations were confirmed by DNA sequencing. For FRET and TIRF microscopy experiments, HEK293-H cells were transfected with the indicated CFP and YFP constructs (100 ng each per coverslip). For electrophysiology, the indicated STIM1 constructs were transfected into HEK293-H cells either alone (200 ng DNA per coverslip) or together with Orai1 (100 ng Orai1 and 500 ng STIM1 DNA per coverslip). All transfections were performed using Lipofectamine 2000 (Thermo Fisher Scientific) 24 to 48 h prior to electrophysiology or imaging experiments.

### Solutions and chemicals

The standard extracellular Ringer’s solution used for the FRET experiments contained 150 mM NaCl, 4.5 mM KCl, 2 mM CaCl_2_, 1 mM MgCl_2_, 10 mM tetraethylammonium chloride (TEA-Cl), 10 mM D-glucose, and 5 mM Hepes (pH 7.4 with NaOH). The Ca^2+^-free extracellular solution contained the following: 150 mM NaCl, 4.5 mM KCl, 3 mM MgCl_2_, 1 mM EGTA, 10 mM TEA-Cl, 10 mM D-glucose, and 5 mM Hepes (pH 7.4 with NaOH). For the electrophysiological studies, the extracellular Ringer’s solution contained 20 mM CaCl_2_, 130 mM NaCl, 4.5 mM KCl, 10 mM TEA-Cl, 10 mM D-glucose, and 5 mM Hepes (pH 7.4 with NaOH). The internal solution for the patch-clamp studies contained the following: 135 mM Cs aspartate, 8 mM MgCl_2_, 8 mM Cs-BAPTA, and 10 mM Hepes (pH 7.2 with CsOH).

### Electrophysiology

Currents were recorded in the standard whole-cell configuration at room temperature on an Axopatch 200B amplifier (Molecular Devices) interfaced to an ITC-18 input/output board (Instrutech) using Igor Pro software (Wavemetrics) routines for stimulation, data acquisition, and analysis written by R. Lewis (Stanford University). Data are corrected for the liquid junction potential of the pipette solution relative to Ringer’s in the bath (−10 mV). The holding potential was +30 mV. The standard voltage stimulus consisted of a 100-ms step to −100 mV followed by a 100-ms ramp from −100 to +100 mV applied at 1 s intervals. I_CRAC_ was typically activated by passive depletion of ER Ca^2+^ stores by intracellular dialysis of 8 mM BAPTA. All currents were acquired at 5 kHz and low pass filtered with the 1 kHz Bessel filter in the amplifier. All data were corrected for leak currents collected in 100 to 200 μM LaCl_3_.

### FRET microscopy

HEK293-H cells transfected with the indicated CFP- and YFP-tagged STIM and Orai constructs were imaged using wide-field epifluorescence microscopy on an IX71 inverted microscope (Olympus). Cells were imaged with a 60× oil immersion objective (UPlanApo NA 1.40), a 175 W Xenon arc lamp (Sutter), and excitation and emission filter wheels (Sutter). At each time point, three sets of images (CFP, YFP, and FRET) were captured on a cooled EM-CCD camera (Hamamatsu) using optical filters specific for the three images as previously described. Image acquisition and analysis was performed with SlideBook software (Imaging Innovations Inc). Images were captured at exposures of 100 to 500 ms with 1 × 1 binning. Lamp output was attenuated to 25% by a 0.6 ND filter in the light path to minimize photobleaching. All experiments were performed at room temperature.

FRET analysis was performed as previously described (13). The microscope-specific bleed-through constants *a* and *d* were determined from cells expressing cytosolic CFP or YFP alone. The apparent FRET efficiency was calculated from background-subtracted images using the formalism (40):

$$E_{FRET}=\frac{F_{c}}{F_{c}+GI_{DD}}$$

where *F*_*c*_ = *I*_*DA*_ − *aI*_*AA*_ − *dI*_*DD*_

*I*_*DD*_, *I*_*AA*_, and *I*_*DA*_ refer to the background subtracted CFP, YFP, and FRET images, respectively. The instrument-dependent *G* factor was determined periodically as described (41). E-FRET analysis was restricted to cells with YFP/CFP ratios in the range of 2 to 6 to ensure that E-FRET was compared across identical acceptor to donor ratios.

### TIRF microscopy

HEK293 cells expressing the indicated STIM1-YFP mutants and CFP-Orai1 were illuminated by laser output from an Argon-ion laser (Melles Griot; 457–514 nm output) coupled to an illuminator that focused light on the back focal plane of a TIRF objective (PlanApo 60×, 1.45 NA, Olympus). TIRF illumination was achieved by controlling the position of a translatable prism to alter the incident angle of the laser beam. CFP, YFP, and FRET images were collected using the following combinations of filters and dichroics (Chroma): CFP excitation: ET458 ± 10 nm; dichroic: Z458/514RPC; emission: ET485/30; YFP excitation: ET514 ± 10 nm; dichroic: Z458/514RPC; emission: ET550 ± 50; FRET excitation: ET458 ± 10 nm; dichroic: Z458/514RPC; emission: ET514 ± 10.

### Image analysis for puncta formation

Batch analysis of puncta was done using a macro written in ImageJ. In brief, a blurred intermediate image was generated using a 2×2 pixels averaging step. Next, the blurred image was subtracted from the original image to enhance the puncta contrast against any diffuse background. The enhanced images were run through an AutoThreshold step using the “Triangle” method. The images were then analyzed using the “Analyze Particle” plugin. The acceptable size of particles was set between 4 and 100 pixels squared (selected based on trial-and-error) to avoid reticular structure artifacts and segment adjacent puncta. The puncta were then counted and saved as individual regions of interest.

The cellular footprint was calculated using a secondary macro. First, a rolling ball background subtraction of 50 pixels in diameter was used to flatten the image. Then using Otsu Threshold, the footprint mask was generated. The “Analyze Particle” plugin was used to measure the cell footprint with a minimum size of 10 pixels. The footprint region of interest was saved and in case the image contained more than one cell, the total cellular footprint was calculated.

### Statistical analysis

All bar graphs summarizing data are represented as mean ± SEM. Individual points are always indicative of biological replicates (distinct cells). Statistical analysis and data analysis was performed using Igor Pro or excel Software. Most comparisons were made using one-way ANOVA followed by a posthoc test (Dunnett or Tukey) as indicated in the Figure legends. Significance is denoted as ∗*p* < 0.05, ∗∗*p* < 0.01, ∗∗∗*p* < 0.001 using posthoc tests as indicated in the legends. In Figure 1, Figure 2, Figure 3, we additionally used a stringent threshold equal to the E-FRET value of the WT construct in the store-depleted state + 2 × SD of the WT dataset for flagging mutants as “hits”. This value was employed as the threshold at which mutants of the screen were assigned as positive “hits”. We note that statistical analysis using one-way ANOVA followed by Dunnett posthoc test with a significance of *p* = 0.005 yielded the same mutants as the hits determined from the threshold level described above.

## Data availability

All of the data shown are contained within the article.

## Supporting information

This article contains supporting information.

## Conflict of interest

The authors declare that they have no conflict of interest with the contents of this article.
