# Supplementary material for: Mapping interactions between the CRAC activation domain and CC1 regulating the activity of the ER Ca2+ sensor STIM1
Source: J Biol Chem. 2022 Jun 17;298(8):102157. doi: 10.1016/j.jbc.2022.102157 (PMC9304783; doi:10.1016/j.jbc.2022.102157)
Supplement: Supplemental Figures S1 and S2 [file mmc1.pdf]

Supplementary Data

Mapping interactions between the CRAC activation domain and CC1 regulating the activity of the ER Ca^{2+} sensor STIM1

Nisha Shrestha, Ann Hye-Ryong Shim, Mohammad Mehdi Maneshi, Priscilla See-Wai Yeung, Megumi Yamashita, and Murali Prakriya

Department of Pharmacology, Northwestern University Feinberg School of Medicine, Chicago, IL 60611

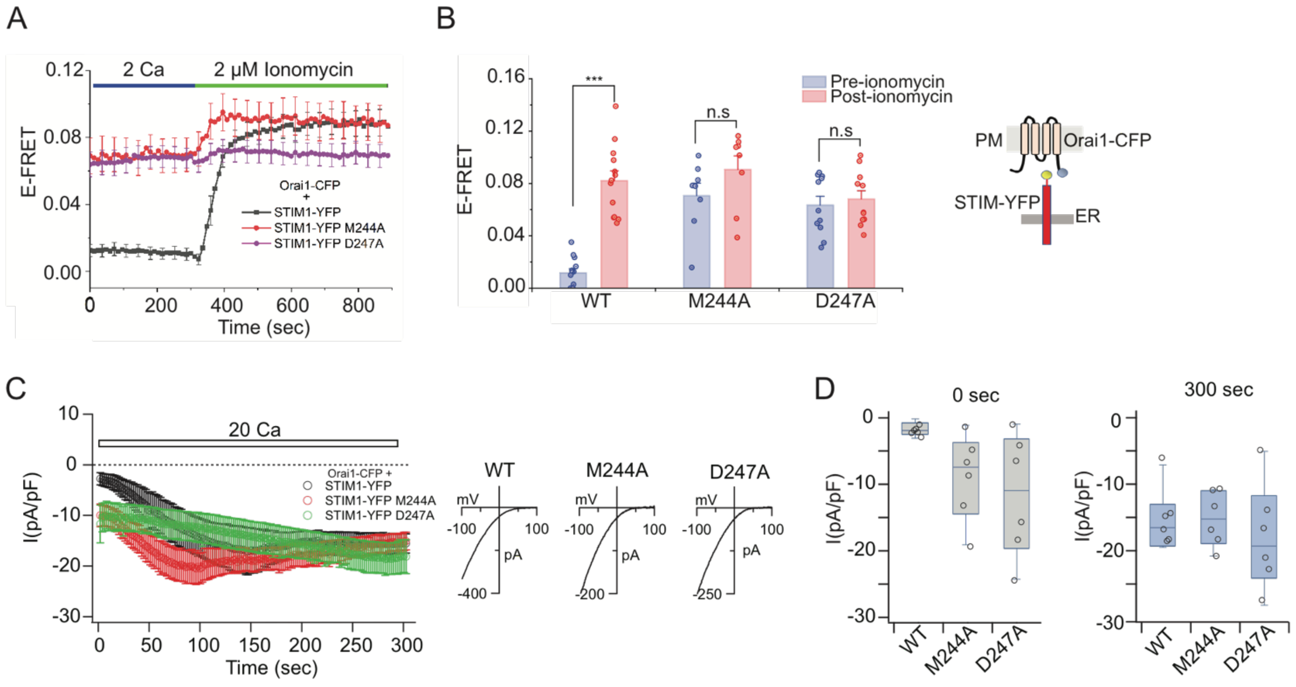

Supplementary Figure 1. The M244A and D247A mutations in CC1α1 cause constitutive activation of STIM1 and CRAC channels. (A) Time-lapse traces of E-FRET between STIM1-YFP and Orai1-CFP in the indicated STIM1 mutants. ER Ca^{2+} stores were depleted by administration of 2 μM ionomycin in a Ca^{2+} -free Ringer's solution. M244A and D247A STIM1 show high levels of resting FRET relative to WT STIM1, indicating constitutive binding of STIM1 to Orai1. **(B)** Summary of the E-FRET values from individual cells in the indicated STIM1 variants at rest and 300 s following store depletion. ***: p:0.001 by unpaired T-test. n ≥ 4 cells. **(C)** STIM1 M244A and D247A cause constitutive activation of CRAC currents. I_{CRAC} was recorded from HEK293 cells co-expressing Orai1-CFP with the indicated STIM1 variants. ER Ca^{2+} stores were passively depleted with 8 mM BAPTA dialyzed into the cells and the traces show the time course profile of the development of CRAC currents following whole-cell break-in. The current-voltage relationship of the activated CRAC currents at t=300 s is shown in the right plots. Please note that the WT time course and I-V traces shown in these panels are the same as the WT currents examples shown in Figure 4C. **(D)** Summary of the CRAC currents amplitudes at time t=0 s and 300 s following whole-cell break-in.

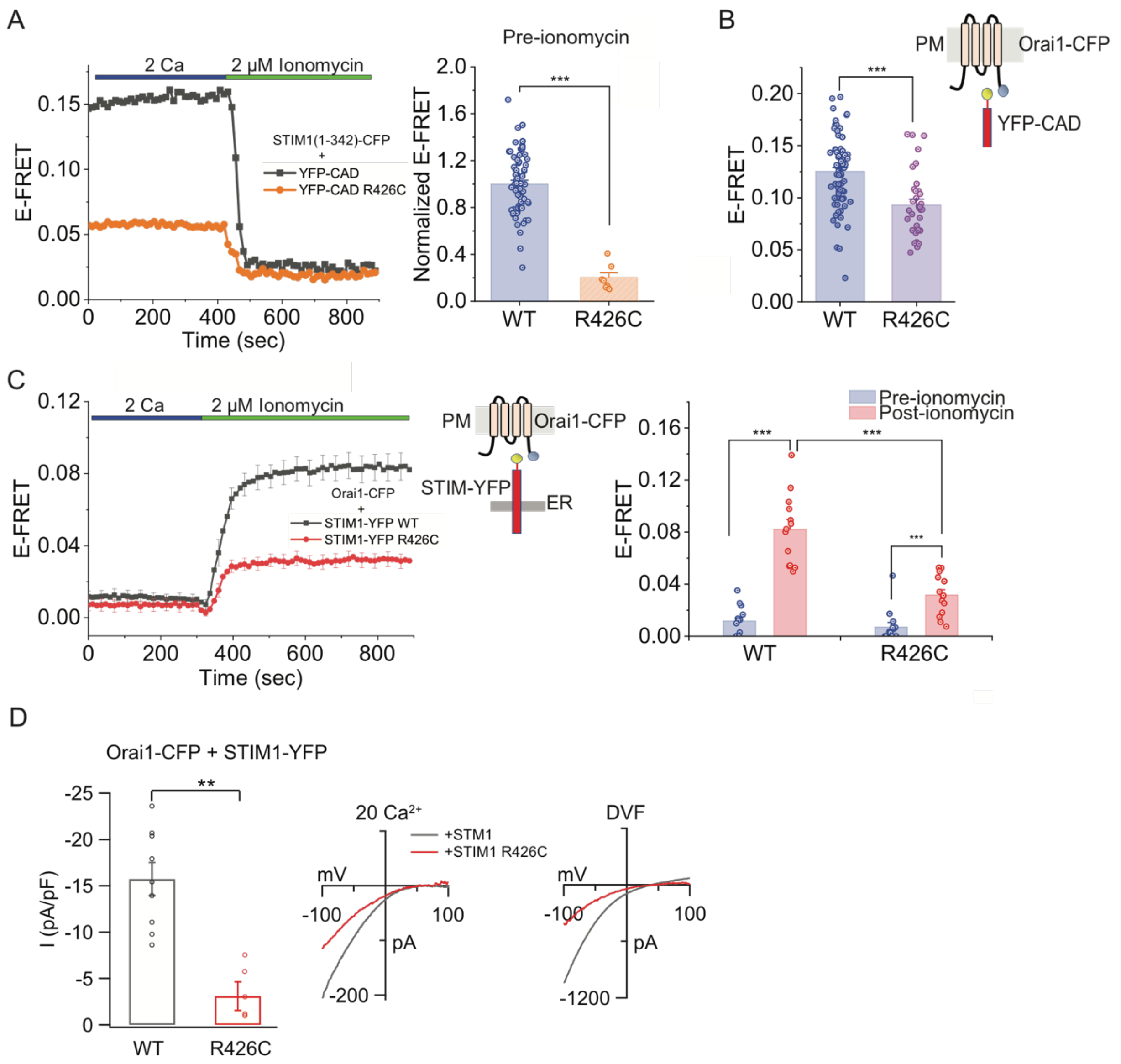

Supplementary Figure 2. Functional analysis of the disease-linked R426C mutation. **(A)** Example E-FRET traces using the 2-component system (Fig. 1) of $STIM1_{1\text{-}342}$-CFP with YFP-CAD and YFP-CAD R426C. Resting E-FRET between $STIM1_{1\text{-}342}$-CFP and YFP-CAD R426C is significantly reduced. The bar graph on the right summarizes the E-FRET data across multiple cells (n ≥ 4 cells). P<0.001 by Unpaired t-test. **(B)** The R426C mutation diminishes E-FRET between Orai1-CFP and YFP-CAD. (n≥ 4). P< 0.001 by unpaired T-test. **(C)** The R426C mutation impairs interaction of full-length STIM1 with Orai1. Example traces of the time-dependent changes in E-FRET between Orai1-CFP and STIM1-YFP. Store depletion causes association of Orai1 with STIM1 which is reduced in STIM1 R426C. (n≥ 4) P<0.001 by paired t-test within the same cells and unpaired t-tests between WT and R426C STIM1. **(D)** The R426C mutation impairs activation of CRAC currents. I_{CRAC} was recorded from HEK293 cells co-expressing Orai1-CFP with either WT or R426C STIM1-YFP. The left bar graph summarizes the mean current amplitude at t=300 s following whole-cell break-in. n ≥ 4 cells per mutant. **: p<0.01 by unpaired T-test.
